# The Molecular Architecture and Mode of Action of *Clostridium perfringens* ε-Toxin

**DOI:** 10.3390/toxins16040180

**Published:** 2024-04-07

**Authors:** Richard W. Titball

**Affiliations:** Department of Biosciences, University of Exeter, Exeter EX4 4QD, UK; r.w.titball@exeter.ac.uk

**Keywords:** *Clostridium perfringens*, ε-toxin, pore-forming toxin, structure, receptor

## Abstract

*Clostridium perfringens* ε-toxin has long been associated with a severe enterotoxaemia of livestock animals, and more recently, was proposed to play a role in the etiology of multiple sclerosis in humans. The remarkable potency of the toxin has intrigued researchers for many decades, who suggested that this indicated an enzymatic mode of action. Recently, there have been major breakthroughs by finding that it is a pore-forming toxin which shows exquisite specificity for cells bearing the myelin and lymphocyte protein (MAL) receptor. This review details the molecular structures of the toxin, the evidence which identifies MAL as the receptor and the possible roles of other cell membrane components in toxin binding. The information on structure and mode of action has allowed the functions of individual amino acids to be investigated and has led to the creation of mutants with reduced toxicity that could serve as vaccines. In spite of this progress, there are still a number of key questions around the mode of action of the toxin which need to be further investigated.

## 1. Introduction

*Clostridium perfringens* ε-toxin is produced by biotype B and D strains of the bacterium. These biotypes are associated with enteritis and enterotoxaemia in young livestock animals and especially in goats, calves and foals [1]. Enterotoxaemia in livestock is often fatal and death can occur soon after the first signs of disease [2]. The infection is typically acquired after the animal ingests spores of the bacterium, which then germinate in the gut. Disease is also associated with a dysbiosis in the gut, often as the result of a change in diet, which allows the multiplication of *C. perfringens* and the subsequent production of ε-toxin. The toxin crosses the gut wall, enters the bloodstream and then binds to target tissues. Histological analysis of ligated intestinal loops of sheep and goats exposed to ε-toxin showed necrosis of the colonic epithelium, and this may change the permeability of the intestine, allowing toxin translocation, possibly by opening tight junctions in the gut mucosa [3,4]. The main target organ is the brain, and entry of the toxin appears to be facilitated by an increase in the permeability of the blood–brain barrier which is associated with damage to vascular endothelial cells [2,5]. Within the brain, cells including neurons, astrocytes and oligodendrocytes are susceptible to ε-toxin [5]. Oligodendrocytes play a role in the myelination of axons, and some researchers have visualised changes to the lamellation of myelin in the brains of diseased animals [6]. Kidney damage is often reported, but it is unclear if this is a direct or an indirect consequence of disease caused by ε-toxin [7].

The ε-toxin gene (*etx*) is located on plasmids in both biotype B and D strains of *C. perfringens* [8] and the *etx* sequence, encoding a 311 amino acid protein, was first reported in 1992. There is no significant sequence homology between ε-toxin and sequences in the NCBI database [9]. The toxin is secreted as a relatively inactive 296 amino acid prototoxin [10], which is activated by proteolytic cleavage, for example by digestive proteases such as trypsin or chymotrypsin [11] or by *C. perfringens* λ-protease [12,13]. Proteolytic activation releases N- and C-terminal pro-peptides, with a consequent increase in toxicity of between 80- and 1000-fold [14,15]. The site of cleavage differs from protease to protease, and individual proteases may cleave at multiple sites. Therefore, the activated toxin may exist in a number of isoforms [14,15]. Whilst it was originally believed that the removal of both the N- and C-terminal pro-peptides was required for activation, a study which characterised recombinant forms of the toxin lacking the N- or C-terminal pro-peptides showed that activation is largely a consequence of the removal of the C-terminal pro-peptide [15]. It is not clear what the biological function of ε-toxin is in relation to the bacterium. However, like tetanus and botulinum toxin, it is possible that the potency reflects the benefit of rapidly killing the host and creating a local environment which is enriched for organic materials and then allows the proliferation of *C. perfringens*.

The mode of action of the toxin has, in the past, been a topic for speculation. In mice, the potency of the toxin, exceeded only by tetanus and botulinum toxins, was originally believed to indicate an enzymatic activity. The hope that gene cloning and sequencing would reveal a possible mode of action was dashed because ε-toxin did not show significant sequence homology to other toxins, or indeed to other proteins. Similarly, work to identify the receptor for ε-toxin led to diverse theories about the nature of the receptor molecule(s), and attempts to identity the receptor were, until recently, unsuccessful.

This manuscript reviews the major advances that have been made in our understanding of the mode of action and molecular basis of the toxicity of ε-toxin. These advances have provided new insight into this toxin, challenged the idea that enzymatic activity is necessary for high potency, renewed interest in the potential role of the toxin in multiple sclerosis (MS) in humans and led to new vaccine candidates for the prevention of disease caused by ε-toxin.

## 2. *C. perfringens* ε-Toxin Is a Member of the Aerolysin Family of Pore-Forming Toxins

**Figure 1:**
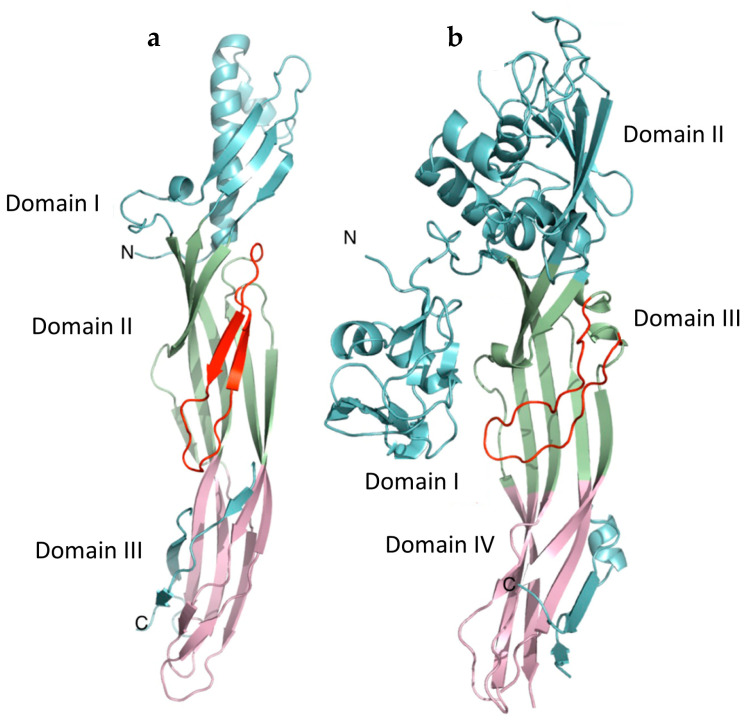
Comparison of the molecular structures of ε-toxin (**a**) and aerolysin (**b**). N- and C-termini of the proteins are marked. The domains are coloured according to function, with receptor-binding domains shaded cyan and the pore-forming modules composed from domains shaded green and pink. The amphipathic loop which forms the inner part of the transmembrane β-barrel is shaded red and located in domain II in ε-toxin and domain III in aerolysin. The C-terminal pro-peptide, removed by proteolytic cleavage is also shaded cyan. Figure modified from [17].

The molecular structure of the monomeric form of ε-toxin was first reported in 2004 [9] and revealed an elongated (100 Å × 20 Å × 20 Å) molecule with three domains, composed mainly from β-sheet (Figure 1a). Although there was no significant sequence similarity with aerolysin, a pore-forming toxin produced by *Aeromonas hydrophila*, the overall structures of ε-toxin [9] and aerolysin [16] showed similarities. However, ε-toxin comprises three domains, while there are four in aerolysin (Figure 1). This finding suggests that ε-toxin is a member of the aerolysin family of β-pore-forming proteins. This family includes proteins from diverse origins (Table 1) which show similarity of structure and mode of action, although there is little amino acid sequence homology between them. Proteins belonging to the aerolysin family are typically secreted as water-soluble monomers which bind to receptors on target cells and oligomerise to form ion-selective pores in the target cell membrane. Much of our understanding of structure–function relationships in members of this group are based on the pioneering studies with aerolysin, the prototypic member of this group. Bacterial toxins in the aerolysin family, such as ε-toxin, aerolysin, monalysin and the binary larvicide AB toxin (BinA/BinB) play a role in the etiology of microbial diseases in hosts ranging from insects to fish and mammals. Eukaryotic proteins in the aerolysin family, such as lysenin and *Laetiporus sulphureus* lectin, may be host defence proteins.

Although proteins in the aerolysin family show some similarities, there are important differences between the toxins in this group. This has resulted in the proposal for a number of groupings (Table 1), based largely on the differences in the presence and organisation of individual domains and assignment in the pfam database of protein families [18]. *C. perfringens* ε-toxin can be assigned to the ETX/MTX-2 group in the aerolysin family, alongside *Bacillus thuringiensis* Cry51 (Mpp51Aa1), *Bacillus thuringiensis* Cry23 (Mpp23Aa1) and *Bacillus thuringiensis* parasporin-2 proteins [18]. Cry51 and Cry23 are insecticidal toxins, with activity towards coleopterans. Parasporin-2 has attracted attention because of its activity towards cancer cells [19]. The alpha-toxin from *Clostridium septicum* is also a member of the aerolysin group of pore-forming toxins but in the absence of structural information, and a low level of amino acid sequence similarity with ε-toxin, it is unclear whether it is also a member of the ETX/MTX-2 group [20].

Except for monalysin, proteins in the aerolysin family possess a distinct receptor-binding domain (RBD) (Table 1). The possession of a RBD, which is encoded non-contiguously, is a key feature of the ETX/MTX-2 group, and this distinguishes this group from aerolysin which has two RBDs (domains I and II). In the non-Etx, lysenin and Toxin_10 sub-classes, the single RBD is encoded contiguously. In ε-toxin and most other aerolysin family proteins, the RBD is located at towards the N-terminus of the protein, but in lysenin, it is located at the C-terminus. The receptor-binding domains (RBDs) appear to be responsible for the differences in target cell specificity and toxicity, which are reported with different members of the aerolysin family.

All of the proteins possess a pore-forming module (PFM), which can include one or two domains. The PFM includes β-strands and a loop, which is often termed the amphipathic loop and inserts into the target cell membrane to form the inner part of the pore [21]. The amphipathic loop is another conserved feature of aerolysin family proteins. Like ε-toxin, many of the proteins in the aerolysin family are produced as relatively inactive precursors, activated by the proteolytic removal of peptides at the N- or C-terminal. All are presumed to form pores in target cells, and this explains their reported haemolytic activity. However, the number of sub-units forming the pore (where known) ranges from seven to nine monomers. The pore itself is formed from a β-barrel which spans the membrane with a collar at the upper end and a “rivet” which forms on the inside of the membrane and these lock the pore into place [22]. The remainder of this article considers the structural biology of ε-toxin in more detail, making comparisons where appropriate with data available for other toxins in the aerolysin family.

## 3. The Receptor-Binding Domain

Human red cells, but not red cells from sheep, goat, mouse, rat, cow, dog, monkey, rabbit or horse are susceptible to lysis by ε-toxin [23]. In contrast, aerolysin causes the lysis of erythrocytes from a broad range of species, with the rat cells susceptible to sub-nanomolar concentrations [24]. Most cell lines tested are resistant to ε-toxin [25]. Some are highly sensitive to the toxin, for example Madin Darby Canine Kidney (MDCK) cells [26], with a CT_50_ dose of 1–10 nM [27,28] and MOLT-4 human T cells with a CT_50_ dose of 11 nM [28]. Aerolysin is active towards a broad range of cell lines, and when tested using a sensitive cell line, aerolysin is a more potent cytolytic agent than ε-toxin with EL4 T-cells killed by concentrations as low as 10 pM [24]. However, aerolysin and ε-toxin differ markedly in their toxicity to small mammals. The lethal dose of aerolysin in mice is approximately 10 µg/kg [29], whereas ε-toxin is at least 100 times more potent [30]. This indicates that the lethality of ε-toxin, compared to that of aerolysin, is not simply a consequence of increased cytotoxicity. Rather, it reflects different target cell specificities.

Whilst the RBDs of ε-toxin (domain I) and aerolysin (domain II) show some general structural similarities, they also show a number of differences. Compared to ε-toxin domain I, aerolysin domain II possesses additional helices, extended loops and two additional strands, resulting in a poor root-mean-square deviation of atomic positions (RMSD) when comparing the structures (1.9 Å over 23 of 62 C-alphas). In aerolysin, domain II is critical for binding to glycan residues on glycosylphosphatidylinositol (GPI)-anchored protein receptors [31] and more specifically, it binds to mannose residues in the glycan core which is tagged onto the lipid moiety of the GPI anchor [32]. Experimental data show that ε-toxin does not bind to GPI-anchored proteins, and although O-linked oligosaccharides have previously been suggested to play a role in the binding of ε-toxin to cells, the protein acceptor for these sugars was not identified in this study [33].

**Figure 2:**
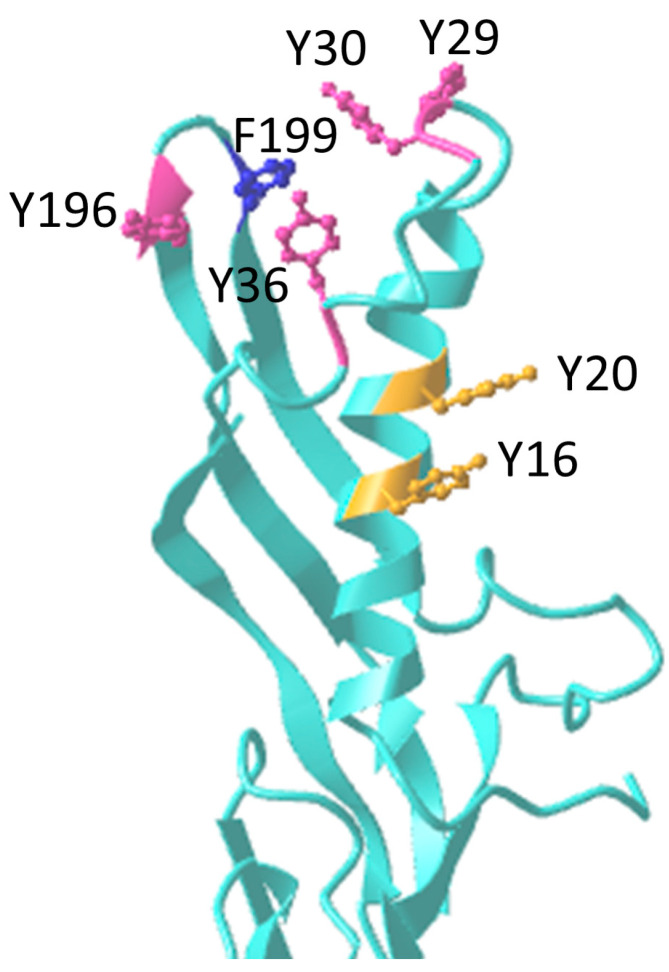
Locations of amino acids in domain I of ε-toxin that are believed to play major (pink) or minor (yellow) roles in binding to receptors. The location of F199 is also shown in blue, though an F199H variant retained toxicity, and an F199E variant appeared to show conformational changes, making it difficult to assess the role of this amino acid in binding.

In ε-toxin, a cluster of aromatic amino acids on the upper face of domain I (Figure 2) appear to be involved in binding to MDCK cells, including Y16, Y20, Y29, Y30, Y36, Y196 and F199 [34,35,36,37]. However, a F199E variant protein showed profound changes in thermal stability compared to wild-type ε-toxin, suggesting significant changes in folding and conformation which may account for the reduction in binding [35,36]. A W199H mutant did not show a significant reduction in toxicity [37]. Therefore, it is unclear if F199 plays a direct role in binding. Amino acids Y16 and Y20 play a minor role in cell recognition [34,36]. The remaining amino acids, Y29, Y30, Y36 and Y196, are located on loops at the top of domain I of ε-toxin, bordering a cleft. This is similar in location and organisation to the cleft in domain II of aerolysin, which has been shown to be the site of mannose-6-phosphate binding in the glycan core on CD52, a GPI-anchored protein [32]. A recent modelling study by Kumar et al. [38] has suggested additional aromatic amino acids of ε-toxin toxin that may play a role in binding, and this now requires testing experimentally. The side chains of aromatic amino acids are often involved in binding to carbohydrate-binding proteins [39]. It is possible that the side chains of Y29, Y30, Y36 and Y196 of ε-toxin stack against the pyranose rings of sugars. However, other members of the aerolysin family use tyrosines to bind to the amino acid side chains of protein [40] or sphingomyelin receptors [41].

Whilst domain I clearly plays a key role in receptor binding by ε-toxin, a possible β-octyl glucoside binding site has also been identified in domain III [34]. In the pore form of the toxin, the residues involved in β-octyl glucoside binding [42] are some distance from the outer leaflet of the target cell membrane. The properties of variants with substituted amino acids involved in hydrogen bonding to the glucose unit (E61, V72 and T93) of β-octyl glucoside have not been reported. This makes it difficult to understand the biological significance of this site.

## 4. The Pore-Forming Module

The molecular structures of the monomeric [9] and pore forms [42] of ε-toxin indicate that it is a β-pore-forming toxin, and this is supported by previous biophysical studies which demonstrate the formation of heptameric complexes of monomers in membranes exposed to ε-toxin [15]. Though the pores formed by ε-toxin are sometimes reported to be anion selective [43,44], the molecular structure of the pore shows that there is no overall charge in the lumen of the pore [42]. This is consistent with the finding that both cations and anions can access the pore, with changes in intracellular K^+^, Cl^-^, Ca^2+^ and Na^+^ levels in cells exposed to ε-toxin [44,45,46]. It is believed that these changes in ion concentrations in cells trigger the depletion of cellular ATP, stimulate an AMP-activated protein kinase and cause changes to the permeability of the mitochondrial membrane with translocation of the apoptosis-inducing factor [46].

**Figure 3:**
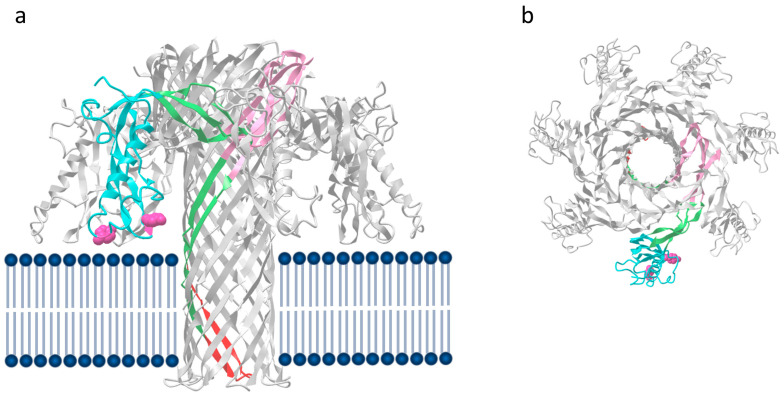
The molecular structure of the ε-toxin pore. A single protomer is shown coloured with domain I cyan, domain 2 pink and domain 3 green. The amphipathic loop in domain 2 is shaded red. The side chains of the receptor-binding residues Y30 and Y196 are show in dark pink. Panel (**a**) The pore is shown in the predicted position in the membrane bilayer (dark blue). (**b**) top view of the pore.

Domains II and III of ε-toxin constitute the pore-forming module, and these domains show structural similarity to domains III and IV of aerolysin. In the ε-toxin monomer, the amphipathic loop that forms the lower part of the transmembrane β-barrel [9] is sandwiched between anti-parallel β-sheets in domain II. However, β-strands flanking the amphipathic loop (residues 95–173) also contribute to the pore [42]. Like aerolysin, it is clear that there are significant differences in the molecular structures of the monomeric [9] and pore [42] forms of ε-toxin (Figure 1a and Figure 3). For aerolysin, the structural data on receptor binding and from mutants trapped at the pre-pore stages have allowed a model to be developed describing changes in the structure during the pore formation process [32]. This prototypic model is valuable for understanding the possible stages involved in pore formation by ε-toxin. An outer β-barrel (residues 63–94, 174–198 and 235–260) forms the so-called collar, surrounding the upper part of the inner β-barrel. Together, the outer β-barrel and the concentric inner β-barrel form the double β-barrel fold, which is characteristic of aerolysin family proteins [21,22,47] (Figure 3).

The ε-toxin pore shows overall structural similarities with the lysenin [48,49] and aerolysin [50] pores, and at its widest point measures approximately 24 Å. At the narrowest point, the diameter is only 12 Å, because of several lysine side chains that occupy the lumen. On the inside of the target cell membrane, T132, V133, P134 and F135 form a β-turn which enables a rivet to form, anchoring the barrel of the pore on the inside of the cell membrane.

The mechanism of pore formation and assembly seems likely to be similar to that of aerolysin. Like aerolysin, ε-toxin forms pores inefficiently in solution, but pores are formed readily on, and in, the target cell membrane. The receptors for both aerolysin and ε-toxin are more abundant in lipid rafts in the cell membrane, and this enables the monomers to bind, become locally concentrated [51] and assemble into structured pre-pores on the cell surface. The structure of the aerolysin pre-pore at different stages has been reported, and this shows an inverted mushroom structure with the stalk extending outwards from the membrane which becomes progressively flattened as the pre-pore moves towards pore formation [32]. The heptamer is believed to insert spontaneously into the lipid bilayer, possibly at the edge of lipid rafts [51]. For ε-toxin, the RBD is believed to remain attached to the receptor during the pre-pore and pore formation processes, and the structure of the RBD changes little, though there is movement of domain I relative to domain II [42]. For aerolysin, the most profound conformational changes in the PFM likely occur as it unfolds and inserts into the bilayer [21], and this also seems likely for ε-toxin.

## 5. Receptors

Whilst aerolysin appears to bind to a wide range of cells, and to a wide range of GPI-anchored proteins in the target cell membrane [51], a number of early studies demonstrated that ε-toxin binds selectively to host cells in vivo. For example, using peroxidase-labelled antibody to ε-toxin Buxton [52] showed that the pro-toxin accumulated in the brain, kidney and liver with some binding to the luminal surface of vascular endothelial cells.

Lipid rafts in cell membranes bring together monomers of the toxin to form the pore [53,54] and some studies implicate lipids such as sulfatides or phosphatidyl serine in binding ε-toxin [54]. This is consistent with the enrichment of these lipids in lipid rafts. Sulfatides are abundant in the myelin sheath, kidney tissues, the gastrointestinal tract and T-cells [55]. Phosphatidylserine is a major phospholipid in the cerebral cortex, though phosphatidyl serine is normally associated with the inner leaflet of the membrane. Cholesterol, which plays a structural role in lipid rafts, is also necessary for the binding of ε-toxin to detergent-resistant membranes [53]. All of this is consistent with ε-toxin binding to lipid rafts. It is possible that ε-toxin can insert into lipid monolayers or bilayers, if the concentration of toxin is sufficiently high to drive multimer formation in the absence of a high-affinity receptor. This would explain why some researchers report that liposomes or monolayers show changes in permeability when exposed to the toxin [46,56,57].

The identity of a protein receptor has been investigated by several groups over the past decades. Unlike aerolysin, the receptor for ε-toxin is not a GPI-anchored protein [45]. Some workers suggested that the receptor is a sialoglyoprotein, since synaptosome membranes treated with pronase or neuraminidase showed reduced binding of the toxin [58].

Two candidate protein receptors have been identified to date (Table 2). The Hepatitis A Virus Receptor (HAVCR1, also known at TIM-1) protein is the receptor for several human pathogenic viruses, and Ivie et al. [59] showed that reducing the expression of HAVCR1 in either MDCK or ACHN cells increased their resistance to ε-toxin. The authors were also able to demonstrate binding of ε-toxin to the extracellular domain of HAVCR1 in vitro [59]. Tyrosine residues in domain I which have been shown to play a role in cell binding (Y29, Y30, Y36 and Y196) appear to play a role in binding to the extracellular domain of HAVCR1 [36]. However, although the expression of HAVCR1 in a toxin-resistant cell line resulted in the expression of the protein on the cell surface, the cells did not bind or become susceptible to ε-toxin. The finding that HAVCR1 expression did not confer toxin sensitivity in cells might indicate that this protein forms part of a receptor complex, but is not the main toxin receptor.

An important advance was made by Rumah et al. in 2015 [60] who showed that CHO cells, which are normally resistant to ε-toxin, become highly sensitive when engineered to express MAL. Moreover, MAL-knockout (Mal^-/-^) mice which no longer express MAL in any cells are highly resistant to intoxication. Also, unlike oligodendrocytes from wild-type mice, oligodendrocytes cultured from Mal^-/-^ mice fail to bind to ε-toxin and are toxin resistant [61]. Subsequently, other researchers have confirmed that the expression of MAL by cultured cells is associated with susceptibility to the toxin [27,28,56,62,63,64]. MAL is associated with lipid rafts in cells, and MAL is reported to oligomerise under some conditions [65]. Evidence of a physical interaction between ε-toxin and MAL was provided by Blanch et al. [28] who showed that the complex could be immunoprecipitated from cells that had previously been exposed to toxin. Collectively, these findings provide a substantial body of evidence that MAL is a major receptor for ε-toxin, but do not preclude an additional role for HAVCR1, lipids or glycans associated with the proteins in binding.

MAL is a 17kDa protein expressed in polarised epithelial cells and in myelin forming cells [66]. MAL is associated with the plasma membrane and also with membranes within cells, and has an essential role in vesicular transport and plasma membrane targeting in a range of cell types [66]. The tissue distribution of MAL does broadly map to the pathology seen in intoxicated animals; it is expressed strongly in epithelial cells in the kidney and in oligodendrocytes which play a role in myelin deposition. It is also expressed in T-cells (especially CD4+ T-cells) and red cells in humans, but not in these cell types in other mammals [66]. In addition to the full-length protein, a number of isoforms exist, and according to a preprint, in humans, an 11kDa isoform is present on red cells which is believed to lack the exon-III encoded region [67].

MAL from different animal species appears to bind to ε-toxin with different affinities. This is well illustrated by comparing the behaviour of CHO cells expressing different MAL; in two independent studies, cells expressing human MAL were at least 10 times less sensitive to ε-toxin than cells expressing rat or sheep MAL [27,60]. More distantly related MAL, for example from zebra fish, appears not to bind to ε-toxin at all, and zebra fish engineered to express human MAL become toxin-sensitive [64]. It also appears that individual amino acids in ε-toxin play different roles in binding to MAL from different species [27,56,60]. The finding that MAL is found on T-cells and red cells in humans but not in other animals indicates that these cells would likely bind to ε-toxin in the blood stream, and this would then limit the amount of toxin that was available to cross the blood–brain barrier and bind to cells in the brain. This might explain why acute toxicity associated with extensive damage to the brain is not seen in humans [68].

The detailed molecular interactions between MAL and ε-toxin are not clear, and there is no structure available of MAL, or the MAL-ε-toxin complex. A study in which the potential interactions between ε-toxin and MAL were modelled [38] might provide information on the interaction, and other studies have provided experimental data. MAL is expressed in a number of isoforms in cells, and the 11kDa isoform of MAL on human red cells [67] would include a binding motif. Rumah et al. [60] have suggested that ε-toxin binds to the predicted extracellular loop 3 of MAL identified by Rubio-Ramos et al. [66], because an insertion into this predicted loop abolished the binding of ε-toxin. However, according to Rubio-Ramos et al. [66], this predicted loop is identical in human and rat MAL, suggesting that it is not responsible for the differences in the susceptibilities of cells expressing human or rat MAL.

Overall, although MAL appears to be an important receptor for ε-toxin, it seems likely that there are other binding partners for ε-toxin, including HAVCR1 and lipids. Possibly, MAL is involved in the transport of HAVCR1 to the cell membrane, and might form a complex with MAL at the cell surface. The observation that ε-toxin binds to endothelial cells lining blood vessels [52], yet MAL is not known to be expressed in normal endothelial cells [66], requires further investigation. Glycans, which may be attached to MAL or HAVCR1, might also play a key role in binding. It is conceivable that different binding partners exist, with molecules such as lipids binding with low affinity, and MAL binding with high affinity to ε-toxin. The determination of the fine molecular structure of the ε-toxin and receptor complex is now an important goal in order to address these questions.

## 6. Exploiting Information on the Mode of Action

In the past two decades, and because of its potency, ε-toxin has received attention as a potential biowarfare or bioterrorism agent. However, a number of findings suggest that the extensive pathology seen in intoxicated animals is less likely to be seen in humans exposed to ε-toxin. There are two reports of the isolation of *C. perfringens* able to produce ε-toxin from the gut of individuals suffering from enteric disease [69]. Neither individual was reported to show signs of intoxication, and there was no report of death as a consequence of the infection [69]. Also, as highlighted above, the pathogenesis of disease in humans caused by ε-toxin would likely be different to that seen in animals because of differences in MAL distribution and affinity for the toxin.

However, it is possible that epsilon toxin causes chronic disease in humans, and a paper published by Murrell in 1986 [70] first outlined the hypothesis that there was a link between exposure to ε-toxin and the later development of MS in humans. This hypothesis was largely based around the observation that five of eight researchers who had worked on swayback disease (an ovine neurological disease) developed MS [71], while the probability of this chance happening was 1 in 10^9^. Murrell considered that these researchers could have been exposed to *C. perfringens* strains which produced ε-toxin. In a broader epidemiological study, Murrell confirmed an association between exposure to sheep or sheep products and the development of MS [70]. However, he could not find antibodies to ε-toxin in individuals who developed MS. More recently, the finding that the receptor for ε-toxin is MAL, which is located in the myelin sheath of neurons, triggered renewed interest in this hypothesis. Subsequently, antibodies which react with ε-toxin in MS patients have been reported [72,73]. Using the PCR, two separate studies [74,75] have shown that the *etx* gene is found at higher frequencies in faecal samples from MS patients compared to matched controls (61% vs. 13% and 14% vs. 3% in the two studies). Isolates cultured from MS patients have been shown to produce functional ε-toxin [72,75]. Moreover, when present, the abundance of *C. perfringens* strains which possess the *etx* gene was higher in MS patients than in controls [75]. There was no pathology reported in control subjects who tested positive for *etx*. It is possible that individuals become colonised after ingesting foodstuffs, such as unpasteurised milk, that contains *etx*-positive *C. perfringens* [76]. Using the experimental autoimmune encephalomyelitis model of MS researchers showed that ε-toxin might play a role in triggering the disease by opening the blood–brain barrier [75]. Overall, the possibility that ε-toxin plays a role in MS warrants further research.

Information on the structure of ε-toxin is being exploited in several ways. Variant proteins with reduced abilities to bind to cells, located in domain I, are one route to the development of low-toxicity variants that could be exploited as vaccines [27,37,77,78,79,80]. The mutation of other residues, for example F118 located in the transmembrane loop [81], also results in a reduction in toxicity. Also, the targeted mutation of selected residues to prolines [82] can reduce toxicity, but this may be because the substitution disrupts the structure of the toxin rather than because the amino acid being targeted plays a key role in toxicity. The mutation of H149 and F92 residues within or surrounding the β-octyl glucoside binding site results in a modest reduction in toxicity [34,83]. Other residues such as Y71 [83,84], which is located in domain III, have also been targeted for mutagenesis and may play a role in pore formation.

An alternative approach to devising a vaccine against pore-forming toxins has been proposed by Hu et al. [85] working with *Staphylococcus aureus* α-hemolysin. This involved allowing the toxin to bind to nanoparticles coated with host cell membrane to generate “nanotoxoids”. It is unclear if the bound toxin formed pores, but the resultant nanoparticles did not show toxicity or reversion to toxicity. Similar findings have been reported with *C. perfringens* ε-toxin [86]. The nanoparticles coated with *S. aureus* α-hemolysin were immunogenic, eliciting neutralising antibody, but it is not clear if this was antibody directed against the monomer or the pore forms of the toxin, and whether antibody was able to neutralise toxicity after the toxin had bound to target cell membranes. The finding that the structures of the monomer and pore forms of the toxin are markedly different suggests the limited conservation of B-cell epitopes and highlights the need to understand more fully how neutralising antibodies can protect against pore-forming toxins.

A longer-term goal might be to engineer the properties of the pore for applications in biotechnology or medicine. Knapp et al. [46] have reported that targeted amino acid substitutions to the amino acid lining the pore can change the selectivity of the pore towards cations. Interestingly, the ion-selectivity of the pore correlated poorly with cytotoxicity.

## 7. Conclusions

The past decade has seen major advances in our understanding of the molecular basis of the toxicity of ε-toxin. *C. perfringens* ε-toxin is one of the most potent bacterial toxins known, exceeded only by botulinum and tetanus toxin. For many years, this high toxicity was thought to indicate an enzymatic mode of action. We now know that ε-toxin does not have an enzymatic mode of action, and its potency is a consequence of targeted damage to cells bearing the myelin and lymphocyte protein (MAL) receptor. It is also clear that, like other pore-forming toxins, remarkable changes in the structure of the protein take place as it becomes bound to and then inserts into target cell membranes. However, we do not know the detail of the intermediate steps in the pore-forming process.

This finding might open opportunities to engineer the toxin with different target cell specificities, to selectively kill some cells or to enhance drug delivery into cells. We are now close to understanding how the toxin interacts with membranes, but it is not clear if MAL alone can serve as a receptor, or whether other membrane components are required, and if so, what role they play in binding. Understanding the interaction between ε-toxin and MAL is a priority to address this question. The finding that MAL appears to be the major receptor protein has renewed the interest in the possible role of ε-toxin in MS, and additional work is required to investigate this possibility. This is a significant challenge because animals other than humans are not known to develop MS, and the distribution and affinity of receptors is different in humans compared to other mammals.

## Figures and Tables

**Table 1:** Comparison of epsilon toxin with selected membrane active proteins belonging to the aerolysin group of pore-forming proteins.

Sub-Class	ETX/MTX-2	Aerolysin	Non-Etx	Lysenin	Monalysin	Toxin_10
Example	Epsilon-toxin	Aerolysin	Laetiporus sulphureus lectin	Lysenin	Monalysin	BinA/BinB (Tpp1/Tpp2)
Origin	*C. perfringens* (Gram-positive bacterium)	*A. hydrophila* (Gram-negative bacterium)	*Laetiporus Sulphureus* (edible mushroom)	*Eisenia fetida* (earthworm)	*Pseudomonas entomophila* (Gram-negative bacterium)	*Lysinibacillus sphaericus* (Gram-positive bacterium)
Disease associations	Enterotoxaemia in animals. (MS in humans)	Motile Aeromonas Septicaemia in fish. Enteric disease in humans	Reported anticancer properties; used in traditional medicine	Believed to be a host defence protein	Lethal infection of insects	Lethal to some mosquito larvae species. Potential anti-cancer agent
Organisation	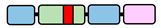	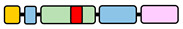	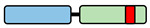	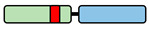	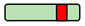	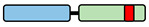
Pfam	PF03318	PF01117	PF03318	Not assigned	Not assigned	PF15431
Monomers forming pore	7	7	Not known	9	Not known	Not known
Receptor	Myelin and lymphocyte protein (MAL)	GPI-anchored proteins	N-acetyllactosamine	Sphingomyelin	Not reported	Cpm1/Cqm1 amylomaltase (GPI anchored protein)
Activation	N- and C-terminal peptide removal	C-terminal peptide removal	Not reported	Not reported	N-terminal peptide removal	Insect gut proteases
Haemolytic activity	Haemolytic only for human red cells	Haemolytic towards a range of mammalian red cells.	Haemolytic towards a range of mammalian red cells	Haemolytic towards a range of mammalian red cells	Haemolytic to human red cells	Not reported
Toxicity in animals	LD_50_ ~50 ng/kg in mice	LD_50_ ~10 μg/kg in mice	Not lethal to zebrafish embryos at 600 μg/mL	Coelomic fluid containing lysenin is toxic to some vertebrates	Lethal to *Drosophila* sp.	Lethal to *Culex* sp.

Graphics (not to scale) show the linear organisation of domains in the proteins. Receptor-binding domain is shown in cyan and orange. Pore-forming module (pale green and pink) containing the amphipathic loop (red). In the case of epsilon toxin and aerolysin, the receptor-binding domain is composed from two non-contiguous regions.

**Table 2:** Comparison of HAVCR1 and MAL as receptors for epsilon toxin.

Property	Protein Receptor	
	HAVCR1	MAL
Tissue distribution corelates with susceptibility to toxin?	No. HAVCR1 is expressed in all organs. Some resistant cells do not express HAVCR1. Some sensitive cells express HAVCR1	Yes, except normal endothelial cells do not express MAL
Membrane localization	NR	In lipid rafts
Expression in cells confers toxin-sensitive phenotype?	No	Yes
Reduced expression increases resistance to toxin?	Yes	Yes
Gene inactivation confers whole animal resistance to toxin?	NR	Yes, in mice
Physical interaction of toxin with protein demonstrated?	Yes	Yes
ε-toxin amino acids involved in receptor binding	Y29, Y30, Y36, Y196	Y30, Y196

NR = not reported.

## Data Availability

Data reported in this review can be found in the relevant cited article, available at https://pubmed.ncbi.nlm.nih.gov/ (accessed on 12 January 2024).
